# Guideline recommendations for diagnosis and clinical management of Ring14 syndrome—first report of an ad hoc task force

**DOI:** 10.1186/s13023-017-0606-4

**Published:** 2017-04-11

**Authors:** Berardo Rinaldi, Alessandro Vaisfeld, Sergio Amarri, Chiara Baldo, Giuseppe Gobbi, Pamela Magini, Erto Melli, Giovanni Neri, Francesca Novara, Tommaso Pippucci, Romana Rizzi, Annarosa Soresina, Laura Zampini, Orsetta Zuffardi, Marco Crimi

**Affiliations:** 1 Department of Molecular Medicine, University of Pavia, Pavia, Italy; 2 Institute of Genomic Medicine, Catholic University School of Medicine, Rome, Italy; 3 Pediatrics Unit, Department of Women’s and Children’s Health, IRCCS Arcispedale Santa Maria Nuova, Reggio Emilia, Italy; 4 Laboratory of Human Genetics, Galliera Hospital, Genoa, Italy; 5 Child Neurology Unit, IRCCS Istituto delle Scienze Neurologiche, Bologna, Italy; 6 Medical Genetics Unit, Department of Medical and Surgical Sciences, S. Orsola-Malpighi University Hospital, Bologna, Italy; 7 Ospedale S. Anna, Ambulatorio Oculistica, AUSL di Reggio Emilia, Reggio Emilia, Italy; 8 Medical Genetics Unit, Department of Woman, Child and Urologic Diseases, S. Orsola-Malpighi University Hospital, Bologna, Italy; 9 Neurology Unit, Department of Neuro-Motor Diseases, IRCCS Arcispedale Santa Maria Nuova, Reggio Emilia, Italy; 10 Unit of Pediatric Immunology, Department of Pediatrics, University of Brescia, ASST Spedali Civili di Brescia, Brescia, Italy; 11 Department of Psychology, University of Milano-Bicocca, Milan, Italy; 12 Ring14 International, Scientific office, Via Flavio Gioia, 5-42124, Reggio Emilia, Italy

**Keywords:** Ring14 syndrome, Recommendations, Caregivers, Best practices, Autistic behavior, Intellectual disability, Seizures, Feeding difficulties, Absent speech, Autism, Malnutrition, Global developmental delay, Growth delay, Abnormality of the face, Abnormality of the retina, Stereotypy, Aggressive behavior, Hyperactivity, Behavioral abnormality, Intellectual disability, Focal seizures, Myoclonus, Hypertelorism, Full cheeks, Horizontal eyebrow, Large forehead, Blepharophimosis, Strabismus, Underdeveloped supraorbital ridges, Thin vermilion border, Epicanthus, Facial asymmetry, Downslanted palpebral fissures, Myopia, Abnormality of retinal pigmentation, Retinal degeneration, Abnormality of the eye, Cataract, Optic neuropathy, Glaucoma, Cafe-au-lait spot, Ventriculomegaly, Short stature, Osteoporosis, Microcephaly, Respiratory tract infection, Abnormality of the corpus callosum, Muscular hypotonia, Celiac disease, Arthritis, Scoliosis, Recurrent infections, Brain atrophy, Osteopenia, Autoimmunity, Status epilepticus, Fever, Encephalopathy, Flexion contracture, Dehydration, Hearing impairment, Recurrent pneumonia, Recurrent upper respiratory tract infections, Pneumonia, Abnormality of vision, Pallor, Astigmatism, Abnormality of skin pigmentation, Respiratory insufficiency, Aspiration, Abnormality of the immune system, Diaphragmatic weakness, Respiratory failure, Dysphagia, Anorexia, Increased body weight, Constipation, Pain, Milia, Focal seizures with impairment of consciousness or awareness, Coloboma, Microphthalmia

## Abstract

**Background:**

Ring chromosome 14 syndrome is a rare chromosomal disorder characterized by early onset refractory epilepsy, intellectual disability, autism spectrum disorder and a number of diverse health issues.

**Results:**

The aim of this work is to provide recommendations for the diagnosis and management of persons affected by ring chromosome 14 syndrome based on evidence from literature and experience of health professionals from different medical backgrounds who have followed for several years subjects affected by ring chromosome 14 syndrome. The literature search was performed in 2016. Original papers, meta-analyses, reviews, books and guidelines were reviewed and final recommendations were reached by consensus.

**Conclusion:**

Conventional cytogenetics is the primary tool to identify a ring chromosome. Children with a terminal deletion of chromosome 14q ascertained by molecular karyotyping (CGH/SNP array) should be tested secondarily by conventional cytogenetics for the presence of a ring chromosome. Early diagnosis should be pursued in order to provide medical and social assistance by a multidisciplinary team. Clinical investigations, including neurophysiology for epilepsy, should be performed at the diagnosis and within the follow-up. Following the diagnosis, patients and relatives/caregivers should receive regular care for health and social issues. Epilepsy should be treated from the onset with anticonvulsive therapy. Likewise, feeding difficulties should be treated according to need. Nutritional assessment is recommended for all patients and nutritional support for malnourishment can include gastrostomy feeding in selected cases. Presence of autistic traits should be carefully evaluated. Many patients with ring chromosome 14 syndrome are nonverbal and thus maintaining their ability to communicate is always essential; every effort should be made to preserve their autonomy.

## Background

This project was undertaken in behalf of, and in direct collaboration with, Ring14 International (R14I), a non-profit worldwide organization supporting and networking medical and scientific activities for the benefit of persons affected with syndromes involving chromosome 14 (www.ring14.org) [1], with the aim of providing guidelines for the management of persons diagnosed with these syndromes. Our guidelines are based on peer-reviewed scientific literature and on the personal experience of professionals representing different areas of medical and social expertise. Since available references on the Ring chromosome 14 syndrome (r(14) syndrome) are quite sparse, consensus was reached through a slightly modified Delphi process [2], which allows agreement based on guide practice when published evidence is lacking.

### Introduction on ring chromosomes

Ring chromosomes belong to the group of structural aberrations of chromosomes, conferring a ring-shaped appearance instead of their normal stick-shaped morphology. Such an alteration has been reported for every human chromosome, although almost half of them affect acrocentric chromosomes (13, 14, 15, 21, and 22). In most cases ring chromosomes are generated by small terminal deletions in both the short and the long arm followed by fusion of the two ends. Intact rings have been reported, especially when conventional cytogenetics was the only diagnostic tool, likely preventing the detection of cryptic genomic imbalances. It must be noted that for some ring chromosomes, a duplication contiguous to the terminal deletion has been described, most likely deriving from the breakage of a dicentric chromosome [3]. A main feature of ring chromosomes is their mitotic instability due to the occurrence of a sister chromatid exchange that can result in the formation of a dicentric chromosome [4]. The instability of dicentric chromosomes leads to different cellular division outcomes, which include cells lacking all or parts of the original ring chromosome, cells containing a double ring, or cells carrying more than one ring. Except rare cases, ring chromosomes occur *de novo*, although it is not clear whether they occur in gametes or in post-zygotic cells [5]. Cote [6] and Kosztolányi [7] described the so-called ring-chromosome syndrome, which is characterized by growth retardation, psychomotor delay of varying degrees and other minor anomalies, regardless of the chromosome involved. According to these authors, ring syndrome is the result of ring instability, which prevents a proper cellular division and increases cellular mortality.

### Ring chromosome 14 syndrome

R(14) syndrome (OMIM #616606) is extremely rare and its prevalence and incidence are unknown. To date, over 80 cases have been described since the first report in 1971 [8]. The size of the terminal deleted region is variable and ranges from less than one to a few megabases. Furthermore, in some individuals, complex rearrangements combining deletions to partial duplication of 14q have been observed [9, 10]. Ring instability is common, as demonstrated by the finding in conventional cytogenetics of cells which are monosomic for chromosome 14 and cells with a double ring or with two rings [11]. The percentage of aneuploid cells varies among patients, usually below the sensitivity of a CGH-array [12]. In fact, at least in blood, cells harboring the ring are the prevalent line, accounting for no less than 85–90%. Clinical features of r(14) syndrome generally include peculiar facial dysmorphisms, psychomotor delay, occasional retinal abnormalities, and intractable epilepsy [13, 14]. Some features of r(14) syndrome are shared with 14q32q33 deletion syndrome, even though with a less degree of severity for the latter [15].

### Pathophysiology

The rarity of the r(14) syndrome, the size variability of the duplication/deletion, the occurrence of mosaicism, and other not yet fully elucidated mechanisms have so far prevented precise genotype-phenotype correlations [16]. However, haploinsufficiency caused by deletions is not an exhaustive explanation for the pathogenesis of r(14) syndrome, since the terminal deletion identified in many subjects is a common gain/loss copy number variant in the normal population [17]. In fact, the evidence that the clinical phenotype of ring carriers is usually more severe than that observed in patients with similar linear 14q terminal deletions suggests that the ring conformation might produce perturbation of the epigenetic state of specific genes along chromosome 14 [18]. In this sense, recent studies have confirmed gene expression changes in patients with r(14) syndrome, providing a significant clue on the effects of the ring formation [19]. Among the possible mechanisms responsible for the perturbation of the epigenetic state, heterochromatin spreading and possible repositioning of the ring chromosome inside the cell nucleus have been prospected [20].

## Symptoms and major complications

### Developmental delay and intellectual disability

One consistent feature of individuals affected by r(14) syndrome is moderate to severe intellectual disability [21]. This variable impairment does not appear to depend on the presence or absence of deletion or on its length, but rather on the time of onset and severity of epileptic seizures: an earlier onset is related to higher degree of disability and to the presence of possible autistic traits [22]. Postural control is achieved on average by the first year, while first steps take place between 2 and 3 years, although there are individuals with r(14) syndrome who never acquire the ability to walk. Language is one of the most affected developmental area. Behavior disorders, including hyperactivity and motoric stereotypies, were found in a large proportion of patients [23]. Children with r(14) syndrome are usually good natured and only occasional bursts of aggressiveness have been reported [21]. Autistic traits, including stereotyped or repetitive motor movements, inflexible adherence to routines, hyper- or hypo-reactivity to sensory input, may be present [24]. In some individuals, the administration of the Autism Diagnostic Observation Schedule (ADOS) revealed a full autistic phenotype [25].

### Epilepsy

All individuals with r(14) syndrome suffer from epilepsy, which is drug resistant in most cases. Onset is variable, but usually occurs in the first months of life [26, 27]. Seizures may be either primarily generalized, tonic-clonic, myoclonic-tonic and clonic or focal starting from the temporal or frontal lobe with a secondary generalization. In more recent literature it has been well demonstrated that seizures are rather focal seizures with or without secondary generalization [28]. Typically, seizures occur upon awakening or falling asleep and can occur in clusters, lasting a day or more. Status epilepticus (seizures lasting more than 5 min) has been reported in about half of the individuals. The course of epilepsy is rather typical, starting with frequent severe seizures that may ameliorate over time and eventually decrease in frequency during late adolescence [21, 29]. It has been also noted that an intense epileptic activity, more frequent in the early years of life, may be associated with regression of the stages of psychomotor development or previously acquired language, suggesting a possible encephalopathic effect of epilepsy [30]. In r(14) syndrome, EEG is characterized by slow and unstructured background activity both during sleep and in awakening. These are associated with discharges of slow, large and asynchronous waves, predominant in the frontal or medio-posterior regions or with complex spike and wave derivations in the occipital region [31]. However, any specific EEG pattern has been proved to be associated with this syndrome.

### Facial features

The facial aspect of children affected by the r(14) syndrome has recurring common features. There can be a various combination of long and sometimes asymmetrical face, full cheeks, large forehead, hypoplastic supraorbital ridges with horizontal eyebrows, strabismus, apparent hypertelorism, epicanthal folds, blepharophimosis, and down-slanting palpebral fissures. The nose usually has a flattened root and bulbous tip and the philtrum may be elongated. The ears may be low-set and the mouth is small with very thin vermillion borders, and downturned corners [32]. All these features have been reported only in patients with r(14) syndrome carrying a long arm terminal deletion of at least 650 kb. The correlation between facial phenotype and 14q terminal deletion is confirmed by the presence of these characteristics in patients with pure linear deletions [33].

### Ocular anomalies

The ocular phenotype in r(14) syndrome includes a wide range of manifestations. In many patients myopia, strabismus, cataracts, maculopathy, optic nerve damage, glaucoma and anomalies in retinal pigmentation have been reported [21, 34]. On the other hand, in a small number of patients, microphtalmia and colobomas have been described as well [35].

### Other clinical manifestations

Recurring features include postnatal-onset microcephaly, hypotonia, scoliosis and increased incidence of infections, particularly respiratory infections, which may require hospitalization [32, 36]. A susceptibility to infections was also observed in patients with linear deletions, suggesting that the phenomenon is due primarily to haploinsufficiency as the distal region of 14q contains the genes for heavy chains of antibodies [21, 37]. Only some individuals with r(14) syndrome have altered immunological and/or antibody profile. The severity of these infectious episodes is variable, spanning from recurrent upper airways infections to severe pneumonia [26, 32, 38]. Other less frequently observed signs and symptoms are short stature, sleep disorders, celiac disease, juvenile osteoporosis/osteopenia, arthritis and cafe-au-lait spots. Gastrointestinal symptoms and malnutrition affect many children, as in most severe neurocognitive disorders. Congenital malformations (heart, kidneys and urinary tract) are very rarely reported. Magnetic resonance imaging does not typically detect any specific changes. In some cases, nonspecific signs such as mild brain atrophy, abnormalities of the corpus callosum, mild ventricular dilatation, hippocampus anomalies and structural alterations of the cerebellum have been observed [32, 39].

### Clinical evolution and prognosis

A precise prognosis in terms of expected lifespan in individuals with r(14) syndrome has yet to be established. Considering the significant clinical variability, it is clear that prognosis should be estimated individually and depends primarily on comorbidities and medical complications. In this sense, the epileptic burden, infectious complications and nutritional deficiencies should be considered as major determinants. A definite cure is not yet available; obviously, all medical efforts should be addressed to treat symptoms, improve life quality and prevent as much as possible observed complications.

## Method for reaching consensus

Our guidelines are based on data from the peer-reviewed scientific literature and on a subsequent holistic summary by a heterogeneous panel of experts. Our methodology was based on three basic steps:

- generation of a list of preliminary recommendations by the core group (BR, AV, and MC) a restricted cluster of authors selected among the panelists;
- based on these recommendations and evidence from literature, the panel drafted a set of guidelines (“Recommendations”) for diagnosis and follow-up; the guidelines were discussed in detail before final approval;
- the document was evaluated by the Scientific Advisory Board of R14I and finally approved by the Board of Directors.

Initially, the core group performed a comprehensive search undertaken to collect the most appropriate published papers on r(14) syndrome. MEDLINE-OVID and the Science Citation Index (ISI) were the most searched databases, from 1971 to 2016. The entry terms employed were: “chromosome ring 14”, “ring chromosome 14”, “ring 14 syndrome”, “ring 14 chromosome”, “chromosome ring 14 syndrome”, “ring chromosome 14 syndrome”. Of all the papers gathered from public databases (more than 150 original articles), the core group have selected those that dealt with the following topics: 1) insights in the formation of the r(14); 2) contribution to the possible pathological mechanisms; 3) report and definition of the clinical traits of the r(14) syndrome; 4) elements of clinical and social assistance. Afterwards, the core group identified a list of seven main areas to be deepen in this paper: 1) Diagnosis, 2) General management and follow-up, 3) Treatment of the major symptoms, 4) Respiratory issues, 5) Nutritional support, 6) Communication and language disorders, and 7) Scientific research aspects. The “Treatment of the major symptoms” topic was further divided into four classes of recommendations: Epilepsy, Hypotonia, Recurrent infections, and Vision and Hearing. The panel of experts, which includes clinical and molecular geneticists, neurologists, immunologists, psychologists and researchers, received from the core group the initial list of recommendations on the topics listed above. Based on bibliographic knowledge and their personal experience, participants were asked whether they agreed, disagreed (being asked to provide a reason) or were unable to comment on each of them. The findings were scored according to a rating scheme adopted by the European Federation of Neurological Societies [40] resulting in three recommendation grade levels: A (definitely useful/strong literature), B (possibly useful/modest literature) or C (good clinical practice but no literature available). Moreover, detailed references were also requested. Even if consensus was achieved, comments were still considered and incorporated in order to better clarify the recommendation. The panel provided inputs through a Delphi process [2], which has been acknowledged as a valid technique for reaching consensus while guaranteeing inclusivity, relative anonymity and economy. There were three rounds for participants to comment on the developing recommendations. For each round, only the recommendations that had been altered in the previous one were considered. For altered recommendations, participants received detailed explanation of why the statement had been changed.

## Results

### Nomenclature

Since its first description the r(14) syndrome has been named with different terminology. We propose henceforward to refer to the OMIM nomenclature, i.e., “ring chromosome 14 syndrome”.

#### Diagnosis

The diagnosis of r(14) syndrome can be so far achieved only through cytogenetic tests: currently no clinical, biochemical, electrophysiological and neuro-radiological data have been found to be specific nor determinant for the diagnosis.

***When to suspect it?*** Actually, r(14) syndrome has a variable and subtle phenotype, common to many other conditions, so that it is hard to suspect the syndrome. ***Which tests to choose?*** As stated in general guidelines for indications to molecular and conventional cytogenetic investigations, children with neuro-psychological alterations and drug-resistant epilepsy, are usually addressed to array-CGH analysis [41] as first diagnostic step (Table 1). All subject for whom a 14q terminal deletion is identified should be addressed to a standard karyotype to assess the presence of the ring. On the contrary, if any genomic imbalance is detected, conventional cytogenetics should be taken into account in the diagnostic process, so that even for those rare individuals carrying the r(14) chromosome with not-detectable deletion the correct diagnosis may be reached [42].

**Table 1:** Diagnosing r(14) syndrome: recommended investigations

Tests	Recommended tests
Clinical chemistry	1st line laboratory test (blood counts, glucose, liver function, CPK, uric acid), T4, TSH, and T3.
Radiology	Cerebral MR (after karyotyping evaluation)
Ophthalmology	Fundus oculi, electrophysiological examination (pev, erg)
Genetics/molecular	• Array-CGH • Conventional karyotyping
Neuropsychology	Complete neuropsychological evaluation

### Differential diagnoses

R(14) syndrome has a severe predominantly neurological symptomatology, common to many genetic conditions. It is important to point out that linear terminal deletions of the long arm of chromosome 14 can be associated with pathological phenotypes, constituting the 14q32 deletion syndrome. The main difference between the two syndromes is the more frequent occurrence of epilepsy in patients with r(14) syndrome.

### Communicating the diagnosis

Communicating a r(14) syndrome diagnosis to parents requires specific skills and abilities. If not performed appropriately, the effect can be shocking, leaving the caregivers with a sense of abandonment and despair. Specialized multidisciplinary clinics (tertiary centers) can provide optimized diagnostic and management services for children with r(14) and their families.

***Recommendation 1.1)*** *The diagnosis should be pursued as soon as possible: children with neurodevelopmental disorders are usually addressed to conventional or molecular cytogenetic tests. Karyotype analysis is essential for the detection of the ring chromosome and the definite diagnosis of r(14) syndrome:* ***Grade A***

***Recommendation 1.2)*** *Karyotype: The analysis of at least 30 metaphases is necessary for a >95% chance of detecting a r(14) chromosome that occurs in at least 80% of cells:* ***Grade A***

***Recommendation 1.3)*** *Patients with r(14) syndrome should consult with an experienced geneticist with the highest priority:* ***Grade A***

***Recommendation 1.4)*** *The diagnosis should be communicated in person by a geneticist, ensuring enough time for discussion with the parents ensuring to provide sufficient and clinically detailed information and avoiding unwanted information:* ***Grade C***

***Recommendation 1.5)*** *Provide printed materials about the r(14) syndrome, R14I, health care services and useful websites. A copy of the clinical report can be helpful for any caregivers and must be authorized by parents. Assure parents that they will be supported by a professional social-clinical team, with regular follow -up visits, ideally within the next 6 months:* ***Grade C***

#### General management and follow-up

The management of children with disabilities is one of the important challenges that pediatricians are currently facing. These patients often require a multi-disciplinary approach which takes into account all the medical issues of the child. Patients and families should be supported by a healthcare services and a professional care team with regular follow-up visits (Recommendation 2.4).

***Recommendation 2.1)*** *Multidisciplinary care at a major clinical facility should be available for patients and their relatives to reduce medical complications and improve quality of life. The general practitioner could have consultation from the following specialists: child neurologist, respiratory physician & therapist, gastroenterologist/nutritionist, dietitian, pediatrician, cardiologist, rehabilitation medicine physician, social counsellor, occupational therapist, speech therapist, specialized nurse, physical therapist, ophthalmologist, immunologist, and psychologist:* ***Grade C***

***Recommendation 2.2)*** *The initial clinical evaluation should comprehend: cerebral MR, EEG, heart and abdomen US, oculistic and audiologic evaluation, neuropsychological assessment:* ***Grade C***

***Recommendation 2.3)*** *Other common examinations usually performed in the r(14) syndrome are: celiac disease and autoimmunity screening, first tier immunological analysis, auxological evaluation:* ***Grade C***

***Recommendation 2.4)*** *Affected children should generally be reviewed with the following scheme: Immunologist, once per year; child Neurologist/Neurologist, as needed; Ophthalmologist, as needed; Gastroenterologist/Nutritionist, as needed and in case of malnutrition; Rehabilitation therapists (physical, psychomotor, speech and language therapist), every week if needed:* ***Grade C***

***Recommendation 2.5)*** *The patient’s support team should keep regular contacts with the family between visits. A referent of the support team should be in charge of keeping contacts:* ***Grade C***

***Recommendation 2.6)*** *Ideally, an effective coordination is essential between the multidisciplinary clinical team, the general practitioner and primary community services:* ***Grade C***

***Recommendation 2.7)*** *Parents and caregivers should preserve as much autonomy as possible for their child:* ***Grade C***

***Recommendation 2.8)*** *Good relationship between patients and others caregivers is vital; they also may have health needs: psychological support should be considered and offered to all caregivers when necessary:* ***Grade C***

#### Symptomatic and specific treatment of the major symptoms

##### Epilepsy

Epilepsy is undoubtedly the most characteristic clinical manifestation of r(14) syndrome. Recurrent infectious episodes, more or less associated with fever, often increase the recurrence of seizures [43]. Seizure frequency tends to decrease over time but only in exceptional cases they completely disappear [44]. General recommendations during seizures: do not attempt physical containment, note the type of seizure and measure duration, and monitor patients for signs of hypoxia in case of seizures associated with significant oxygen desaturation (in which case oxygen therapy may be needed). Antiepileptic drugs are frequently used: barbiturates (in the first months of life), valproic acid, carbamazepine, topiramate, vigabatrin, clobazam and levetiracetam. About half of patients with r(14) syndrome show drug resistance, while in the remaining half seizure control is variable. The Ketogenic diet or Vagal Nerve Stimulation may be taken in consideration for the therapeutic strategy [45]. Interrupting effective antiepileptic therapies when the symptomatology appears less severe could lead to the return of seizures that may even result no longer responsive to previously effective treatments.

##### Hypotonia

Hypotonia is quite common in the r(14) syndrome. Reduction of muscle tone can be generalized or involve predominantly axial muscles. Hypotonia tends to decrease gradually over time, also depending on the rehabilitation. Hypotonia has a dampening effect on motor development and can often lead to scoliosis and/or flat foot. Reduced motility in the early years can cause muscle contractions and/or tendinous retractions.

##### Recurrent infections

Over their entire life, many individuals with r(14) syndrome suffer from frequent occurrence of infections of the upper respiratory tract, while cases of recurrent pneumonia are rarer. Only some individuals with Ring14 have altered immunological and/or antibody profile [37]. The severity of these infectious episodes is variable, spanning from recurrent upper airways infections to severe pneumonia. As for other categories of fragile individuals, clinical presentation can be unusual, with initial non-specific deterioration of general condition (e.g., the late occurrence of fever), followed by tumultuous progression. Comorbidities, such as epilepsy and scarce nutrition, may dramatically worsen clinical management. Since infections are one of the worse prognostic factors and the main cause of death in adulthood, all measures preventing infections must be undertaken: high hygiene standards, parental training for early diagnosis of infections, extensive and possibly extended vaccination plans, precocious hospitalization.

##### Vision and hearing

Refractive errors (such as myopia and astigmatism) and/or retinal degeneration, associated with pigmentary abnormalities, can be frequently observed in r(14) syndrome. Several complications can be detected by a fundus oculi evaluation, such as thinning of vessels, sub-atrophy with pale appearance of the optic disk, depigmented areas, hypochromic spots or widespread irregularities of the retinal epithelium. Strabismus is another important condition [35].

***Recommendation 3.1)*** *Conduct regular clinical follow-up examinations to evaluate epileptic seizures:* ***Grade A***

***Recommendation 3.2)*** *Do not interrupt effective antiepileptic therapies even if the symptomatology appears less severe:* ***Grade C***

***Recommendation 3.3)*** *Start physical therapy early to reduce hypotonic complications (such as scoliosis) due to the reduced muscle tone:* ***Grade B***

***Recommendation 3.4)*** *Conduct regular physiatric assessments to identify possible hypotonic complications:* ***Grade C***

***Recommendation 3.5)*** *Carry out periodic eye examinations with evaluation of the fundus oculi:* ***Grade B***

***Recommendation 3.6)*** *In case of recurrent ear infections, perform audiometric evaluation:* ***Grade A***

#### Respiratory issues

Respiratory complications are frequent in patients with r(14) syndrome, primarily because of diaphragmatic weakness combined with aspiration and recurrent infections. Therefore, it is important to provide a program of respiratory physiotherapy individualized for each patient. Furthermore, when facing with recurrent infections, individuals with r(14) syndrome should be promptly addressed to a clinical immunologist at the first sign of infection to identify possible immunological defect (Table 2) and to optimize therapeutic strategies in compliance with the best standards of care [46] is of the utmost importance. Prompt antibiotic treatment is recommended in all cases to control acute episodes of infection. Antibiotic prophylaxis can also be considered in case of recurrent infection and its duration must be determined in individual cases by the attending physician. To prevent infections, the administration of vaccines is recommended, in particular anti-pneumococcus, anti-H. influenzae, and anti-meningococcal.

**Table 2:** Immunological tests

Serum Immunoglobulins (IgG, IgA, IgM, IgE)	
Lymphocyte sub-populations (CD3, CD4, CD8, CD19, DR, and CD16)	
Adaptive immune system to vaccines such as tetanus, hepatitis	
Complement levels	

***Recommendation 4.1)*** *Any respiratory symptoms should be promptly checked; screening tests for the monitoring of respiratory function are strongly recommended. Signs of respiratory failure should be discussed with the parents to allow advanced planning:* ***Grade A***

***Recommendation 4.2)*** *In case of recurrent pulmonary infections, consider respiratory physiotherapy antibiotic prophylaxis; an immunological evaluation is also strongly recommended:* ***Grade A***

***Recommendation 4.3)*** *Recommended (inactivated) vaccines include anti-haemophilus, anti-pneumococcal, anti-meningococcal and flu vaccine:* ***Grade C***

***Recommendation 4.4)*** *In severe conditions, oxygen therapy at home could be necessary:* ***Grade C***

#### Nutritional support

In most of cases, children with r(14) syndrome are extremely underweight and are frequently affected by anorexia that leads to malnutrition, therefore all children should undergo repeated measures of anthropometric variables and nutritional evaluation. Each clinical visit should include weight measurement and nutrition assessment with proper scales for children with motor impairments. A teamwork with a skilled dietician or nutritionist would then lead to a prescription or oral nutritional supplements when needed. In presence of dysphagia restricted to liquid foods, a speech therapist should be involved to investigate the extent of swallowing disturbance and the possible oral rehabilitation strategy. When oral feeding not possible is only temporarily or in case of severe dysphagia, the use of a naso-gastric tube is the best option. If dysphagia becomes permanent or long lasting, a gastrostomy (PEG) should be considered, either by surgical or endoscopy procedures. PEG is very important to ensure drug delivery, not only for epilepsy, but also for the very frequent gastro-intestinal (GI) symptoms, such dyspepsia and constipation that are respectively treated with anti-acid and osmotic laxative drugs (Table 3).

**Table 3:** Nutritional challenges and suggested standards

Challenges for nutritional issues	Standards of nutritional care
Screening for malnutrition	Anthropometric measures as part of routine clinical assessment
Nutrition support	Managed by a Pediatric nutritional team
Treating malnutrition	Enteral nutrition, oral nutrition supplements and tube feeding as needed, oral rehabilitation

Physicians should note that although quite safe and useful, feeding tubes often intimidate caregivers, that need reassurance and adequate training before starting such treatments. Children with r(14) syndrome have normal digestive and absorptive functions, therefore can be nourished with intact protein formulas and should be allowed to eat all the food they can safely ingest by mouth. Micronutrient and vitamin intake must be monitored; vitamin D daily supplementation must be given at twice the of recommended daily allowance (approximately 800-1.000 IU/day).

***Recommendation 5.1)*** *Nutritional status, especially body weight, should be checked regularly in patients affected by r(14) syndrome:* ***Grade A***

***Recommendation 5.2)*** *Malnourished children should be referred to a pediatric nutrition team:* ***Grade C***

***Recommendation 5.3)*** *Children with dysphagia should be referred to a speech therapist:* ***Grade A***

***Recommendation 5.4)*** *A careful prescription of oral nutritional supplements can help to reduce malnutrition in orally competent children:* ***Grade B***

***Recommendation 5.5)*** *Enteral tube feeding is recommended when anorexia is limiting caloric intake and/or when aspiration occurs during swallowing:* ***Grade A***

#### Communication and language disorders

As reported above, intellectual disability and language disorders have been found in almost all children with r(14) syndrome [22], although high individual variability emerged in their communicative skills. In a survey study was showed that only four out of 12 children and young adults with r(14) syndrome can use words to communicate [23]; in addition, only two of them could speak fluently. It has to be noted that the parents of “non-verbal” children have reported the presence of a higher number of challenging behaviors. Assessment of language development in children with r(14) syndrome has to be conducted by clinicians with sufficient experience not only in language disorders, but also in intellectual disabilities and autism spectrum disorders. Some children seem to use communicative gestures to compensate for their expressive language difficulties [23], so assessment must evaluate also non-verbal communication skills. To increase children’s linguistic abilities, clinical practice suggests the use of augmentative and alternative communication strategies, although there are not yet specific studies on their effective validity in children with r(14) syndrome. The presence of autistic traits in most of these individuals strongly affects their communicative skills. As in children with autism spectrum disorders, an intensive behavioral treatment, combined with parent training, could be helpful in reducing challenging behaviors. A specific program of speech and language therapy has to be planned for every children considering their age and individual characteristics.

***Recommendation 6.1)*** *Full psychomotor and neuropsychological testing is useful from the first 2 years to detect early communication and language disorders:* ***Grade A***

***Recommendation 6.2)*** *Regular assessment of speech and language function by a trained therapist is recommended:* ***Grade B***

***Recommendation 6.3)*** *Communication support systems (*e.g., *pointing boards with figures or words) should be used with individuals with severe communicative impairment; individualized training should be guaranteed to affected people and appropriate support provided to their caregivers:* ***Grade C***

***Recommendation 6.4)*** *If autistic traits are shown, an intensive behavioral treatment, combined with parent training, could be helpful in reducing challenging behaviors:* ***Grade C***

#### Scientific research aspects

In the field of rare diseases, easy access to standardize clinical data [47] as well as to high-quality samples [48] are the key prerequisites for promoting research. Thus, both genetic biobanks and registries/clinical databases play an increasingly important role in facilitating diagnoses and in developing translational research, through collecting, storing and distributing biospecimens and related data in a standardized framework. Therefore, R14I signed in 2009 an agreement with the Telethon Network of Genetic Biobanks (TNGB) in order to provide services for storage and distribution of biospecimens from persons affected by r(14) syndrome and their relatives [49]. The agreement favors a centralized sample collection and ensures visibility via the TNGB online catalogue (http://biobanknetwork.telethon.it/) as well as an easy samples accessibility for a wide scientific community. Request evaluation is carried out in agreement with the board of management of TNGB and its policy based on predefined criteria shared among the partners to avoid any possible conflict of interests with R14I. Moreover, R14I is directly engaged in collecting and storing clinical data of patients and families all around the world in a dedicated and accessible clinical database (http://www.ring14.org/eng/460/database/). Data are directly extracted from clinical records and evaluated by clinicians. Presently more than 300 samples and related data have been distributed to accredited researchers to support project on the r(14) syndrome. Newly diagnosed children and their families are recommended to contribute their data and biological samples to our database and biobank resources. Moreover, R14I is actively funding other scientific projects with dedicated funds available through yearly-issued calls for grants [1]; however, identifying and supporting other areas with further research is urgently needed.

***Recommendation 7.1)*** *Further molecular studies to optimize the treatment of the most relevant symptoms (*i.e., *epileptic crisis and bronchial secretions) are needed:* ***Grade A***

***Recommendation 7.2)*** *Further improvements to harmonize data collection in the clinical database are needed:* ***Grade B***

***Recommendation 7.3)*** *New diagnosed children or families who have not yet participated in clinical database/biobank should be invited to do:* ***Grade B***

***Recommendation 7.4)*** *Further studies on the psychosocial determinants of quality of life in children and caregivers are urgent:* ***Grade C***

***Recommendation 7.5)*** *Further studies to evaluate communication disorders and their treatment in r(14) syndrome are needed:* ***Grade B***

### Unproven therapies

To date, there are no clinical trials and there is no evidence of translational research being currently undertaken to sustain unproven/alternative therapies. However, we have started to collect some preliminary data on the antiepileptic effect of cannabidiol (CBD) on some children affected by r(14) syndrome with severe, intractable, childhood-onset, treatment resistant epilepsy. In a recently published trial study [50], it was reported that CBD doses of 2–5 mg/Kg per day, up-titrated until intolerance or to a maximum of 20–25 mg/kg per day reduced seizure frequency and had an adequate safety profile in children affected by highly treatment-resistant epilepsy. In these cases, the addition of CBD has led to the reduction of the doses of concomitant therapies (especially benzodiazepines). We recommend this as a possible therapy choice for patients with r(14) syndrome, but note that other sources recommend a much lower dosage of CBD (see https://www.aesnet.org/meetings_events/annual_meeting_abstracts/view/2422094).

## Conclusions

The development of best standards and commonly-approved procedures is urgently needed for all rare diseases (www.rarebestpractices.eu) [51]. Indeed, scientific advances on r(14) syndrome and treatment of affected people are the two primary aims in establishing evidence-based guidelines dedicated to clinicians and caregivers. This present effort aims at evaluating existing evidence regarding the diagnosis and clinical management of children with r(14) syndrome. Being a neglected syndrome with a very low incidence, most recommendations are good clinical practice based on the consensus of the participant experts. Further evaluations are needed to improve clinical and social issues of affected children and their families, as well as research recommendations for scientists focused on the r(14) syndrome.

### The point of view of parents

Taking care of a child with a very rare and complex syndrome is challenging. There are many factors to be carefully considered:

- The syndrome is rare and hardly recognizable by family physicians.
- There are many and often overlapping symptoms, some of which are not directly caused by the disease, making it difficult to understand their true origin or cause.
- People with r(14) syndrome cannot express their discomfort and pain due to cognitive and language delays. Parents must observe, interpret, analyze these children and their behavior as objectively as possible in order to correctly explain the health status to physicians with whom it is imperative to create a relationship of mutual trust.
- The disease has a great impact on family life, not only on parents but also on healthy siblings. Often one parent is forced to give up work in order to take care of the sick child, with significant economic and psychological consequences. The attention, time and effort that is dedicated to these very needy children is diverted from the other siblings, who very often experience this situation with uneasiness, even if they love their affected brother or sister and live with the fear of losing them.

In order to care for affected persons, parents need to acquire a certain medical knowledge. They must learn to objectively observe their affected children. However, they must also follow their instincts that allow parents to understand and see things that others may not understand or see (e.g., seizure anticipation). Starting from the diagnosis, parents often become the main reference point of this disease. Through R14I, clinicians now have valuable scientific resources to add to the experience of Ring14 families (who are a very valuable source of knowledge).

### Updates and future revisions

The guidelines and recommendations proposed here will be updated during the 3^rd^ edition of the Ring14 meeting of families and scientists that will be held in 2018. Subsequently, the guidelines will be regularly updated every two years by clinicians selected among the participants of the panel and other healthcare professionals.

## Abbreviations

CBD: Cannabidiol
PEG: Percutaneous endoscopic gastrostomy
R14I: Ring14 International
TNGB: Telethon Network of Genetic Biobanks
